# Pilates Method as a Biopsychosocial Intervention in the Modern Workplace: A Systematic Review of Physical, Mental, and Occupational Benefits

**DOI:** 10.3390/healthcare14131852

**Published:** 2026-06-25

**Authors:** Ioannis Trigonis, Ioannis Tsartsapakis, Aglaia Zafeiroudi, Gerou Maria, Konstantinos Karakatsanis, Gerasimos Grivas, Olga Kouli

**Affiliations:** 1 Department of Physical Education & Sport Science, Democritus University of Thrace, 69100 Komotini, Greece; itrigon@phyed.duth.gr (I.T.); kkarakat@phyed.duth.gr (K.K.); okouli@phyed.duth.gr (O.K.); 2 Department of Physical Education & Sport Science, Aristotle University of Thessaloniki, 62122 Serres, Greece; miamixan@hotmail.com; 3 Department of Physical Education & Sport Science, University of Thessaly, 42100 Trikala, Greece; azafeiroudi@uth.gr; 4 Physical Education and Sports, Division of Humanities and Political Sciences, Hellenic Naval Academy, 18539 Piraeus, Greece; grivasger@hotmail.com

**Keywords:** pilates method, occupational health, workplace wellness, musculoskeletal disorders, biopsychosocial model, employee well-being, physical therapy, mental health

## Abstract

**Highlights:**

**What are the main findings?**

- Evidence indicates that Pilates interventions are associated with reductions in work-related musculoskeletal pain, particularly in the chronic low back and neck pain regions, while contributing to improved core stability.
- The reviewed studies report positive psychosocial outcomes, including trends toward reduced occupational stress and anxiety, alongside enhancements in job satisfaction and professional self-concept.

**What are the implications of the main findings?**

- Pilates appears to be a versatile biopsychosocial tool that can be integrated into diverse workplace settings (onsite, studio, or remote) to support employee well-being.
- Implementing Pilates-based wellness programs may offer a strategic approach to managing occupational health, potentially addressing factors linked to absenteeism and healthcare utilization.

**Abstract:**

**Background/Objectives:** Work-related musculoskeletal disorders (WMSDs) and occupational stress substantially affect workforce health and productivity. This systematic review aimed to synthesize evidence regarding the effectiveness of the Pilates method as a biopsychosocial intervention for employees, examining its impact on physical, psychological, and occupational outcomes. **Methods:** A systematic search was conducted across major electronic databases and search engines (PubMed, Scopus, Web of Science, PEDro, and Google Scholar) following PRISMA 2020 guidelines. The review protocol was prospectively registered in PROSPERO (CRD420261390771). Eligible studies included randomized controlled trials, quasi-experimental, and observational designs involving employees participating in Pilates programs. Outcomes were categorized into three domains: physical health, mental well-being, and occupational performance. **Results:** Twenty-three studies (*n* = 1179 participants) met the inclusion criteria. The evidence indicates that Pilates may reduce pain intensity and disability in workers with chronic low back or neck pain, with moderate certainty based on randomized controlled trials. Improvements in psychological outcomes, including anxiety and job-related stress, were also reported, although the certainty of evidence was lower. Occupational benefits included enhanced job satisfaction and, in limited cases, favorable cost-utility findings. **Conclusions:** Pilates appears to be a feasible multidimensional intervention for workplace health, with potential benefits across physical and psychosocial domains. Further high-quality trials are needed to clarify long-term effects, economic impact, and optimal implementation strategies within occupational settings.

## 1. Introduction

Work-related musculoskeletal disorders (WRMSDs) represent one of the most prevalent and costly occupational health challenges globally, affecting employees across sedentary, manual, and mixed-demand professions. Epidemiological data indicate that WRMSDs account for approximately 30% to 50% of all occupational diseases worldwide, severely impacting global health metrics and imposing an immense economic burden through direct medical costs and lost productivity [1,2,3]. Prolonged sitting, repetitive loading, suboptimal workstation ergonomics, and elevated psychosocial strain contribute to chronic pain, reduced functional capacity, and diminished quality of life [1,2,3]. Office-based workers frequently present with chronic low back pain (CLBP) and neck pain, often accompanied by postural syndromes such as Upper Cross Syndrome (UCS), characterized by forward head posture and thoracic hyperkyphosis [4,5]. Conversely, workers in manual labor sectors, including agriculture and manufacturing, experience distinct biomechanical demands that require mechanistically informed interventions tailored to their occupational exposures [6,7]. These diverse risk profiles underscore the need for accessible, evidence-based strategies capable of addressing both the physical and psychosocial determinants of worker health.

Pilates has emerged as a promising biopsychosocial intervention within this context. As a mind–body exercise system integrating core stabilization, controlled breathing, and neuromuscular re-education, Pilates has been reported in foundational literature to provide meaningful functional improvements across diverse populations [1,2,3,5,8]. The method is fundamentally grounded in six foundational principles: centering, concentration, control, precision, breath, and flow, which collectively function to optimize movement quality and enhance neuromuscular efficiency [9,10]. Biomechanically, the precise integration of these principles targets deep muscular stabilization, specifically increasing the recruitment and thickness of vital trunk stabilizers such as the transversus abdominis, thereby directly counteracting the mechanical deficits induced by prolonged, static occupational postures [10]. Concurrently, from a psychophysiological perspective, the demand for continuous internal focus, mindful somatic awareness, and regulated diaphragmatic breathing establishes a robust cognitive–somatic link; this mechanism serves to downregulate autonomic arousal, thereby mitigating work-related stress, subclinical anxiety, and burnout while actively promoting employee subjective well-being [11].

Mechanistic evidence suggests that Pilates influences both biomechanical and cognitive–affective pathways, including reductions in pain catastrophizing and kinesiophobia [12]. In occupational settings, preliminary evidence derived from randomized controlled trials (RCTs) and pilot studies suggests that interventions of 8–12 weeks are associated with improvements in postural alignment, muscle endurance, and functional capacity [13,14,15,16,17]. Workplace-adapted Pilates protocols may also help address postural deviations characteristic of UCS by strengthening deep cervical flexors and scapular stabilizers [18,19].

The psychosocial dimension of worker health is equally critical. Pilates has been associated with reductions in subclinical symptoms of anxiety, depression, and stress, alongside improvements in vitality, emotional well-being, and sleep quality [11,20,21,22,23,24,25]. Crucially, within the context of occupational wellness, these investigated parameters typically reflect variations in subclinical psychological distress and subjective well-being rather than diagnosed psychiatric disorders classified under the Diagnostic and Statistical Manual of Mental Disorders (DSM-5). During the COVID-19 pandemic, healthcare workers experienced unprecedented psychological strain; preliminary evidence suggests that clinical Pilates, including telerehabilitation formats, may reduce pain and improve mental health in these high-stress professions [23,26]. By enhancing autonomic regulation and promoting muscular relaxation, Pilates may also support restorative sleep and cognitive resilience, both of which are essential for work recovery.

Beyond clinical and psychosocial outcomes, the integration of Pilates into occupational health frameworks carries important economic implications. Musculoskeletal pain is a major driver of productivity losses through absenteeism and, more prominently, presenteeism—working while symptomatic [27,28]. By improving musculoskeletal endurance and psychological resilience, Pilates may mitigate these burdens. Recent budget impact analyses indicate that implementing group-based Pilates can generate substantial societal savings, approximately Int$ 172 million over five years in large-scale systems, primarily through reductions in disability-related costs and productivity losses [27]. These findings position Pilates not only as a therapeutic modality but also as a potentially cost-effective component of occupational health and safety strategies.

Despite the growing body of evidence, no systematic review has comprehensively synthesized the effects of Pilates across the three interconnected domains of occupational health: (1) physical and musculoskeletal function, (2) psychosocial well-being, and (3) ergonomics and economic risk reduction. Existing reviews have typically focused on isolated outcomes or specific clinical populations, limiting their applicability to workplace settings. Therefore, describing the rationale for this review in the context of existing literature highlights the need for a holistic synthesis that bridges these fragmented areas.

The present systematic review aims to synthesize the existing evidence from diverse worker populations and to map the potential of the Pilates method as a comprehensive, biopsychosocial intervention for the modern workplace.

## 2. Materials and Methods

This systematic review was conducted and reported in accordance with the Preferred Reporting Items for Systematic Reviews and Meta-Analyses (PRISMA) updated guidelines. The review protocol was established prior to the execution of the search strategy and was prospectively registered in the PROSPERO International Prospective Register of Systematic Reviews (Registration number: CRD420261390771).

### 2.1. Search Strategy

A comprehensive and systematic literature search was conducted to identify relevant studies investigating the impact of Pilates in the workplace. The search was performed across three major electronic databases (PubMed, Scopus, and PEDro) and supplemented by two comprehensive meta-search engines (Web of Science and Google Scholar). The search retrieved a total of 863 records (PubMed: *n* = 144; Scopus: *n* = 211; Web of Science: *n* = 123; PEDro: *n* = 57; Google Scholar: *n* = 328).

The final search for all databases was conducted on 30 April 2026. No language or publication date restrictions were applied to ensure comprehensive retrieval of evidence. The search strategy utilized a combination of Medical Subject Headings (MeSH) and free text terms. To ensure full reproducibility, the detailed, database-specific search strings tailored to the syntax requirements of each platform are provided in Appendix A.

### 2.2. Eligibility Criteria

To determine the eligibility of the studies, specific inclusion and exclusion criteria were defined based on the PICOS (Population, Intervention, Comparator, Outcomes, Study design) framework, combined with a specific Context (C).

Population: The review included working adults (aged 18–65 years) across various occupational sectors (e.g., corporate office workers, healthcare professionals, industrial/loom workers, and agricultural farmers). Eligible participants included healthy employees participating in workplace health promotion programs, as well as those suffering from or at risk of work-related musculoskeletal disorders (e.g., chronic low back pain, postural misalignments) and psychosocial challenges (e.g., job stress, anxiety, burnout). Studies involving participants under the age of 18, retirees, professional athletes, pregnant women (unless the focus was explicitly on workplace ergonomics), and individuals with severe medical pathologies unrelated to occupational contexts (e.g., acute fractures, malignancies) were excluded.

Intervention and Context: Eligible interventions included any program based on the Pilates method (e.g., Mat work, Reformer, or chair-based modified Pilates) designed for and evaluated within an occupational context. These encompassed interventions delivered on-the-job, online, or via supervised home/studio settings specifically targeting employees. Programs of any duration were accepted. Pilates could be combined with secondary interventions (e.g., ergonomic education, massage) provided that Pilates was the core component. General physical activity programs, yoga, or weightlifting lacking distinct Pilates principles, as well as multimodal programs where Pilates was a minor component, were excluded.

Comparator: Studies utilizing passive control groups (e.g., usual care, wait-list) or active control groups (e.g., alternative physical therapies, McKenzie method, neck stabilization, ergonomic education) were included. For quasi-experimental designs, the participants’ own baseline (pre-intervention) measurements served as the comparator. Studies comparing two different versions of Pilates without a distinct alternative control group or baseline, as well as those using pharmacological or surgical comparators, were excluded.

Study Design: A broad range of quantitative designs was included to capture all relevant evidence. This comprised Randomized Controlled Trials (RCTs), pilot RCTs, non-randomized controlled trials, quasi-experimental studies (e.g., one-group pretest–posttest designs), and structured observational studies. Qualitative studies lacking quantitative data, cross-sectional studies without an intervention phase, case reports (n < 5), narrative/systematic reviews, and conference abstracts lacking detailed methodologies were excluded.

### 2.3. Study Selection and Data Extraction

Following the initial search, all identified records were exported to reference management software where duplicates were removed. Prior to formal screening, the two reviewers piloted the eligibility criteria on a random sample of 50 abstracts to ensure consistent interpretation and refine operational definitions. This calibration step aimed to minimize discrepancies during the main screening phase. Two independent reviewers then screened the titles and abstracts of the remaining records against the eligibility criteria. Studies that appeared relevant underwent a full-text review. All disagreements between the two reviewers at any stage (title/abstract screening, full-text assessment, data extraction, and risk-of-bias evaluation) were resolved through discussion. When consensus could not be reached, a third independent reviewer adjudicated the decision.

Data from the final included studies were systematically extracted using a standardized template. Extracted information included study characteristics (author, year, country, design), participant demographics (sample size, occupation), intervention details (Pilates type, protocol duration, frequency, setting), specific outcome measurement tools, and key findings categorized by evidence pillar (Physical/Musculoskeletal, Mental/Psychological, and Ergonomic/Occupational).

### 2.4. Quality Assessment and Risk of Bias

The methodological quality and risk of bias of the included studies were assessed independently by two reviewers. Due to the methodological heterogeneity of the included studies, two distinct tools were utilized. The Cochrane Risk of Bias 2 (RoB 2) tool was applied to all Randomized Controlled Trials (RCTs), evaluating five domains: the randomization process, deviations from intended interventions, missing outcome data, measurement of the outcome, and selection of the reported result. The Risk Of Bias In Non-randomized Studies of Interventions (ROBINS-I) tool was employed for quasi-experimental and observational studies, assessing seven domains: confounding, selection of participants, classification of interventions, deviations from intended interventions, missing data, measurement of outcomes, and selection of the reported result.

Inter-rater agreement for risk-of-bias assessments was high. Although a formal κ statistic was not calculated, concordance between reviewers was consistently strong across domains. Based on these tools, studies were classified as having an overall “Low”, “Moderate/Some Concerns”, “High/Serious”, or “Critical” risk of bias.

### 2.5. Data Synthesis

A qualitative narrative synthesis was conducted to summarize the findings of the included studies. Although 12 Randomized Controlled Trials (RCTs) were identified, a quantitative meta-analysis was deemed inappropriate due to substantial clinical and methodological heterogeneity. Specifically, the included interventions varied markedly in Pilates delivery (Matwork, Reformer, and chair-based protocols), session frequency (one to five sessions per week), and total duration (4 weeks to 9 months). Additionally, the occupational sectors represented (office-based, healthcare, and manual labor) introduced diverse ergonomic exposures and baseline physical demands. Methodological heterogeneity was also evident in the comparator groups, which ranged from non-active controls and posture education to conventional physical therapy, as well as in the diversity of outcome measures. The use of multiple disparate scales for pain (e.g., VAS, NRS) and disability (e.g., ODI, NDI, RMDQ), coupled with inconsistent reporting of standard deviations or effect sizes in several studies, prevented harmonization into a common effect metric. While the Standardized Mean Difference (SMD) could theoretically address the use of disparate measurement scales, its application was deemed inappropriate given that the extensive clinical heterogeneity, spanning vastly different occupational demands and intervention protocols, would render any pooled effect size statistically unreliable and clinically misleading. Even within pain outcomes, the heterogeneity of measurement tools and reporting formats precluded subgroup meta-analysis. Pooling such heterogeneous data would have produced misleading summary estimates and violated core assumptions of meta-analytic modelling.

Given the mixture of RCTs and quasi-experimental studies, the Synthesis Without Meta-analysis (SWiM) framework provided the most appropriate structure for organizing and transparently reporting the evidence. To ensure a rigorous synthesis, predefined grouping rules were implemented, categorizing data into three primary conceptual domains based on our biopsychosocial framework: (1) physical health and musculoskeletal function (subdivided into pain intensity, muscle endurance, and flexibility), (2) mental and psychological well-being (job-related stress, subclinical anxiety/depression, burnout, and sleep parameters), and (3) occupational performance and ergonomics (presenteeism, absenteeism, and job satisfaction). Within each domain, the direction-of-effect was structured and determined based on the statistical significance (*p* < 0.05) and clinical relevance of the reported post-intervention changes, classifying findings as ‘favorable effect’, ‘no clear effect’, or ‘unfavorable effect’ relative to the respective comparator groups. Furthermore, to specify the outcome metrics used without statistical pooling, individual study effect measures, predominantly absolute mean differences, percentage changes, or native scale scores, were systematically tabulated within comprehensive evidence tables where available, ensuring direct alignment with SWiM reporting guidelines. Due to the qualitative nature of the synthesis and the high heterogeneity, a formal assessment of publication bias via funnel plots was not feasible; however, potential selective reporting was critically appraised within the Risk of Bias framework. In light of these methodological constraints, the synthesis prioritized transparent reporting and structured grouping of outcomes rather than statistical pooling, ensuring full alignment with SWiM recommendations.

## 3. Results

This section presents the comprehensive findings of the systematic review, beginning with a detailed chronological overview of the literature search and study selection process. It further outlines the baseline characteristics of the included literature and provides a structured, qualitative synthesis of the empirical evidence organized around our three primary conceptual pillars: physical and musculoskeletal health, mental and psychological well-being, and ergonomic and occupational performance.

### 3.1. Study Selection

**Figure 1:**
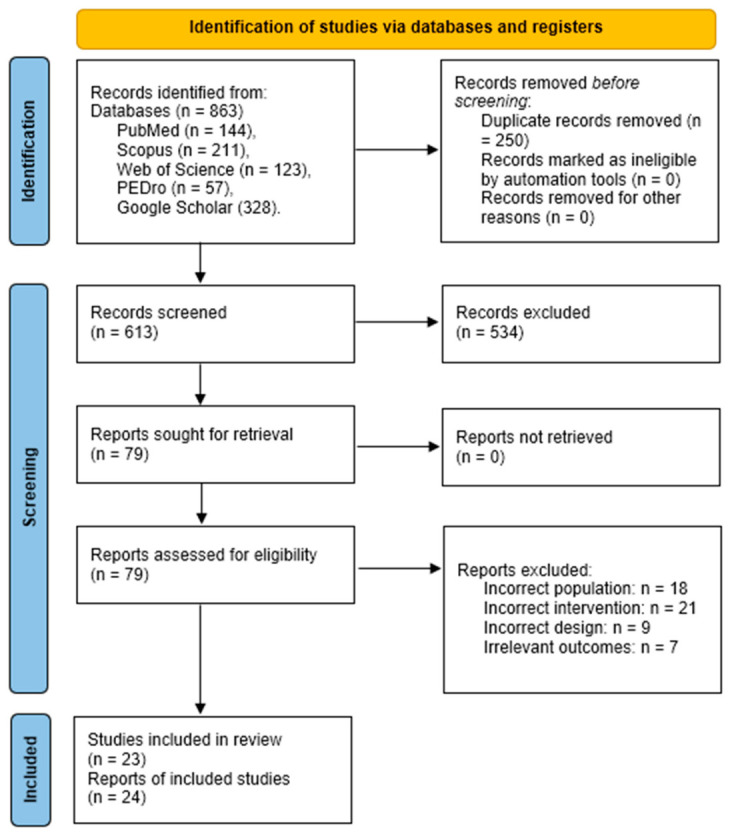
PRISMA 2020 flow diagram.

The systematic search across electronic databases initially identified 863 records. After the removal of 250 duplicates, 613 records were screened based on title and abstract. Of these, 534 were excluded as they did not meet the basic inclusion criteria. The remaining 79 reports were sought for full-text assessment and formally evaluated for eligibility. Following a rigorous eligibility check against the PICO framework, 55 reports were excluded for the following specific reasons: 18 reports involved an incorrect or non-eligible population, 21 lacked a specific or core Pilates-based intervention, 9 utilized an incorrect or non-quantitative study design, and 7 reported irrelevant or missing quantitative outcomes. Ultimately, 23 original studies (represented in 24 publications) were included in the qualitative synthesis. The complete screening process is detailed in the PRISMA 2020 Flow Diagram (Figure 1).

### 3.2. Characteristics of Included Studies

The summary characteristics of the 23 included studies are presented in Table 1. The studies were conducted in diverse geographical regions, reflecting the global interest in workplace Pilates, including Brazil, USA, Italy, Czech, Iran, Germany, Ukraine, Egypt, South Korea, and others. The publication dates range from 2014 to 2026.

Regarding the study populations, the synthesis included a total of 1179 participants (noting that three studies did not specify an exact sample size). The participants spanned various professional sectors, with a high concentration of office workers, healthcare professionals (nurses and hospital staff), and industrial workers. The study designs were predominantly Randomized Controlled Trials (RCTs) and quasi-experimental studies.

### 3.3. Synthesis of Evidence by Pillar

To provide a structured overview of the benefits of Pilates in the occupational context, the findings were categorized into three primary evidence pillars: (1) Physical and Musculoskeletal, (2) Mental and Psychological, and (3) Ergonomic and Occupational outcomes. A summary of the evidence per pillar for each included study is provided in Table 2.

The analysis indicates that Pilates interventions consistently led to significant reductions in pain (particularly chronic low back and neck pain) and improvements in physical function. Psychologically, the studies reported decreased levels of anxiety, depression, and job stress. Furthermore, ergonomic benefits included improved postural alignment and enhanced job satisfaction.

### 3.4. Pilates Intervention Protocols

The specific characteristics of the Pilates protocols, including the method used, duration, frequency, and setting, are detailed in Table 3. The most frequent intervention type was Matwork Pilates, often supplemented with accessories like small balls or magic circles, while Reformer-based programs were also utilized.

Protocol durations typically ranged from 4 to 12 weeks, with a frequency of 2 to 3 sessions per week. A significant portion of the interventions was delivered in a supervised format, either in-person at the workplace or in specialized studios, emphasizing the importance of professional guidance in ensuring exercise quality and safety.

### 3.5. Outcome Mapping and Measurement Tools

The variety of assessment tools employed across the studies to capture the multidimensional impact of Pilates is mapped in Table 4. Pain was most commonly assessed using the Visual Analogue Scale (VAS) or the Numerical Pain Rating Scale (NPRS), while functional disability was frequently measured via the Oswestry Disability Index (ODI) or the Neck Disability Index (NDI). Psychosocial outcomes were captured through validated scales such as the Beck Depression Inventory (BDI) and the Hospital Anxiety and Depression Scale (HADS).

### 3.6. Methodological Quality and Risk of Bias

Finally, the methodological quality of the evidence was evaluated to determine the reliability of the findings. The risk of bias assessment for both randomized and non-randomized studies is presented in Table 5.

While several RCTs demonstrated a low risk of bias, particularly in outcome measurement and reporting, “some concerns” were noted in others regarding the randomization process or the blinding of participants (which is inherently difficult in exercise-based interventions). Non-randomized studies generally exhibited a higher risk of bias, often due to the lack of a control group or potential confounding factors.

### 3.7. Summary of Evidence (GRADE)

A GRADE assessment was conducted to evaluate the certainty of evidence across the core outcomes included in this review. Table 6 summarizes the overall strength of evidence derived exclusively from randomized controlled trials. Across eight RCTs, Pilates interventions consistently demonstrated short-term reductions in pain, yielding moderate-certainty evidence, downgraded for risk of bias and imprecision due to small sample sizes. Similarly, six RCTs reported improvements in functional disability, also resulting in moderate-certainty evidence. Evidence for psychological outcomes (anxiety, depression, stress, kinesiophobia, catastrophizing, sleep) was supported by only two RCTs using heterogeneous measurement tools, including one study with high risk of bias, leading to low-certainty evidence. Finally, QALYs and cost-effectiveness were examined in a single high-quality RCT, providing moderate-certainty evidence, downgraded for imprecision due to single-study data. Overall, the GRADE evaluation indicates that Pilates probably improves pain, disability, and QALYs in working populations, while psychological benefits remain less certain. The detailed rationale for downgrading decisions is presented in the Section GRADE Footnotes.

#### GRADE Footnotes

- Risk of Bias Several RCTs showed “some concerns” in randomization, deviations from intended interventions, or outcome measurement. One psychological-outcome RCT had high risk of bias. This domain was downgraded by one level for pain, disability, and psychological outcomes.
- Inconsistency Effects were generally consistent for pain and disability. Psychological outcomes showed variability due to different measurement tools and populations. This domain was downgraded by one level for psychological outcomes.
- Indirectness No serious indirectness was identified, as all studies involved working adults and assessed relevant occupational or clinical outcomes. No downgrade was applied.
- Imprecision Most RCTs had small sample sizes (<50 participants). Psychological outcomes were supported by only two RCTs, and QALYs were supported by a single RCT. This domain was downgraded by one level for pain, disability, psychological outcomes, and QALYs.
- Publication Bias A formal assessment (e.g., funnel plot) was not feasible due to the absence of a meta-analysis. No downgrade was applied, although potential bias cannot be excluded.

## 4. Discussion

To our knowledge, this is the first systematic review to synthesize the effects of Pilates across three interrelated domains of occupational health: musculoskeletal function, psychosocial well-being, and ergonomic risk. This review of 23 studies involving 1179 participants indicates that Pilates may serve as a versatile biopsychosocial intervention capable of addressing the multifactorial demands of contemporary work environments.

### 4.1. Physical Health and Musculoskeletal Function

Work-related musculoskeletal disorders remain a major contributor to pain, disability, and reduced productivity across both sedentary and physically demanding occupations. The present review demonstrates that Pilates interventions frequently improve core endurance, flexibility, mobility, and pain outcomes. These findings align with recent meta-analytic evidence. Lazoura et al. [38] reported clinically meaningful reductions in chronic neck pain and disability following Pilates compared with non-active controls, with benefits persisting at follow-up. Similarly, de Araujo Cazotti et al. [39] found that Pilates improves function and quality of life in individuals with mechanical neck pain and reduces analgesic use. Beyond spinal outcomes, Pilates appears to enhance systemic physical capacity; Fernández-Rodríguez et al. [40] showed that Pilates improves cardiorespiratory fitness, a key determinant of occupational fatigue.

The applicability of these benefits extends to physically demanding sectors. Perez Cunalata et al. [6] demonstrated that a 12-week Pilates program reduced pain and improved flexibility in poultry workers, suggesting relevance for ergonomically strenuous occupations. Comparable findings have been reported in agricultural settings; Kim et al. [7] observed improvements in body stability and reductions in musculoskeletal symptoms among fruit farmers following a three-month intervention. Collectively, these results support the role of Pilates as a preventive and rehabilitative strategy capable of addressing biomechanical strain across diverse occupational contexts.

### 4.2. Psychosocial Well-Being and Mental Health Mechanisms

Psychosocial stressors, including cognitive overload and organizational pressure, are increasingly recognized as determinants of workplace health. The present review indicates that Pilates may contribute to improvements in emotional stability, optimism, and overall quality of life, while reducing anxiety and somatization. Barbosa et al. [24] demonstrated that a nine-month Pilates program significantly reduced anxiety, depression, and fatigue among bank employees experiencing chronic organizational stress, highlighting its potential as a workplace psychosocial intervention.

When interpreting these psychosocial benefits, a sharper operational definition of the biopsychosocial construct within occupational settings is required. A clear distinction must be maintained between clinical psychiatric conditions and subclinical occupational distress. The improvements observed in anxiety, depression, and burnout scores across the reviewed literature represent shifts in subclinical psychological symptoms and subjective well-being, rather than treatments for diagnosed psychiatric disorders classified under the Diagnostic and Statistical Manual of Mental Disorders (DSM-5) [41,42,43].

Emerging evidence suggests that these benefits may be driven by enhancements in positive psychological functioning rather than large reductions in clinical symptomatology. Meta-analytic findings indicate that Pilates improves quality of life, self-esteem, and vitality, consistent with the pattern observed in workplace-based studies [11]. Mechanistically, mind–body components such as attentional focus, controlled breathing, and body awareness may modulate stress-related symptoms, particularly in occupations characterized by cognitive load and postural strain.

Preliminary evidence from healthcare settings further supports this interpretation. Akyol [23] reported improvements in anxiety, vitality, and mental health among female healthcare workers following remotely delivered Pilates during the COVID-19 pandemic. Although limited by its conference-abstract format, the findings align with broader evidence showing that Pilates benefits both clinical populations [29] and healthy workers. Improvements in vitality and emotional role-functioning suggest that Pilates may influence stress regulation through interoceptive and cognitive–emotional pathways, consistent with contemporary biopsychosocial models of occupational health [44,45].

### 4.3. Ergonomics, Occupational Risk, and Implementation Models

A key strength of Pilates is its adaptability to workplace constraints. The present review supports earlier findings that Pilates can be effectively implemented in settings with limited space or time availability. Haiou et al. [17] demonstrated that chair-based Pilates improved muscle strength, balance, lumbar torque ratios, and pain in office workers compared with posture education, highlighting its feasibility as a micro-break intervention requiring minimal equipment.

For occupations involving high physical strain or psychological burnout, more intensive supervised programs may be necessary. Evidence from the COVID-19 period indicates that online and hybrid Pilates models are feasible and may yield comparable psychosocial outcomes to face-to-face delivery [23,35]. Meta-regression findings further support the equivalence of digital and in-person formats in general adult populations [11], suggesting that remote delivery may be suitable for dispersed or teleworking employees.

Pilates may also address cognitive–behavioral barriers to work recovery. Vera-Saura et al. [46] demonstrated that integrating mind–body cueing into Pilates reduces kinesiophobia in individuals with chronic low back pain, a factor closely linked to presenteeism and delayed return to work. However, heterogeneity in psychosocial responses across non-occupational populations underscores the importance of contextual tailoring. Occupational settings differ widely in ergonomic exposures, job demands, and organizational stressors, suggesting that Pilates may be most effective when embedded within broader ergonomic and organizational frameworks [11].

Furthermore, the synthesized data must be interpreted strictly within the context of the specific occupational cohorts evaluated, such as office workers, healthcare professionals, and manual laborers, who face distinct baseline stressors, avoiding direct extrapolation of these outcomes to the general, non-working population.

### 4.4. Limitations

Several limitations should be acknowledged. A critical methodological caveat of the current literature base pertains to the high occupational heterogeneity characterizing the synthesized cohorts. The included studies evaluated a diverse spectrum of professional groups, ranging from highly sedentary office personnel to high-mobility healthcare workers and manual laborers. Because baseline ergonomic exposures, daily physical demands, and caloric expenditures differ substantially between these environments, the therapeutic and preventative impact of a Pilates program cannot be uniformly extrapolated across all worker categories. Baseline job demands inevitably act as a modulating factor influencing both adherence and clinical efficacy. Consequently, the findings of this review should be interpreted with contextual caution, and future research should prioritize large-scale, industry-specific randomized controlled trials to establish optimized implementation guidelines tailored to distinct occupational risk profiles.

First, although some RCTs demonstrated low risk of bias, many quasi-experimental and observational studies exhibited moderate to serious risk due to non-randomized designs, challenges in blinding exercise interventions, and limited use of active comparators. Second, substantial heterogeneity in Pilates protocols, intervention durations, and outcome measures precluded the possibility of conducting a meta-analysis. Third, evidence directly linking Pilates to long-term economic outcomes, such as absenteeism, presenteeism, or cost–benefit indices, remains limited. Furthermore, while the physical benefits of Pilates are supported by moderate-certainty evidence, the psychological and mental health outcomes currently yield low-certainty evidence according to the GRADE assessment, primarily due to the limited number of RCTs, small sample sizes, and heterogeneous measurement tools. Finally, due to the qualitative nature of the data synthesis and the high methodological heterogeneity, a formal statistical assessment of publication bias via funnel plots was not feasible. Consequently, the presence of selective reporting cannot be entirely ruled out, and the positive trends reported herein should be interpreted with appropriate caution.

### 4.5. Future Directions

The aforementioned limitations identify a major research gap; future studies should prioritize high-quality randomized designs with standardized psychological metrics to clarify the impact of Pilates on workplace-related stress, burnout, and emotional well-being. Future research should prioritize standardized occupational Pilates protocols, including optimal frequency, duration, and exercise modifications for specific professional groups. Large-scale RCTs incorporating explicit cost–benefit analyses and return-on-investment metrics are needed to inform policy and support the integration of Pilates into occupational health and safety guidelines.

### 4.6. Practical Implications for Occupational Health

Workplace Pilates programs may offer a practical, low-cost strategy for improving musculoskeletal function and reducing stress in diverse occupational settings. The adaptability of Pilates to chair-based, on-site, or digital formats supports its integration into existing workplace wellness initiatives with minimal infrastructural demands. Organizations seeking to enhance worker health and productivity may consider incorporating structured Pilates sessions as part of broader ergonomic and psychosocial risk-management frameworks.

## 5. Conclusions

In summary, this systematic review suggests that the workplace application of the Pilates method may function as a potentially effective, multidimensional health-promotion approach. When viewed through a biopsychosocial lens, the modality appears to offer a balanced framework capable of addressing several of the physical and mental demands commonly encountered in modern occupational environments.

From a physical and somatic perspective, the available evidence indicates that Pilates-based interventions can contribute to reductions in work-related musculoskeletal symptoms, particularly chronic low back and neck pain, while also supporting improvements in core stability, postural alignment, and functional capacity. These effects, although not uniform across all studies, were observed with relative consistency in diverse occupational groups.

On a psychosocial level, although the certainty of evidence is generally lower, structured Pilates programs show indications of promoting positive shifts in employee well-being. Reported benefits include reductions in subclinical occupational anxiety, burnout, and perceived stress, together with improvements in job satisfaction, emotional balance, and overall vitality. These findings, however, should be interpreted cautiously given the variability in study designs and measurement tools.

Despite these encouraging trends, the substantial heterogeneity in intervention protocols, program durations, and occupational cohorts underscores the need for careful interpretation. Future research would benefit from moving toward more standardized, industry-specific randomized controlled trials capable of clarifying long-term sustainability, refining implementation strategies, and establishing clearer guidelines for integrating Pilates within occupational health frameworks.

## Figures and Tables

**Table 1:** Summary of characteristics of included studies.

Study (Year, Country)	Population & Sample (n)	Design & Duration	Intervention vs. Comparator	Main Outcomes Evaluated	Pillar
Guidotti et al. [20] (Italy)	Adults 50–64 yrs (n = 49)	OBS, 12 weeks	Matwork Pilates vs. Inactive	Psychological symptoms, Stress behaviors	2
Sabir et al. [28] (Pakistan)	Loom workers with CLBP (n = 46)	RCT, 4 weeks	Pilates Pelvic Tilt vs. McKenzie	Pain, Disability, Pelvic tilt angle	1, 3
Carregaro et al. [27] (Brazil)	Adults with CLBP (n = 145)	RCT, 6 weeks	Group Pilates vs. Home exercises	QALYs, Healthcare costs	3
Hrusova & Komestik [29] (Czech)	Female workers (n = 21)	Q-EXP, 3 months	Modified Pilates vs. Baseline	Spine flexibility (Thomayer, Schober)	1
Karkousha et al. [19] (Egypt)	Females with UCS (n = 40)	RCT, 4 weeks	Pilates vs. Traditional PT	Balance, Spinal curve, Pain, NDI	1, 2
Kolomiitseva et al. [16] (Ukraine)	Female workers (n = 32)	RCT, 12 weeks	Pilates vs. No exercise	Respiratory function, Muscle strength	1
Maan et al. [25] (Pakistan)	Workers with neck pain (n = 52)	RCT, 4 weeks	Pilates vs. Conventional PT	Neck pain, disability, sleep	1, 2
Azam et al. [30] (Pakistan)	Nurses with neck pain (n = 30)	RCT, 4 weeks	Pilates vs. Isometric exercises	Pain, Quality of Life, Kinesiophobia	1, 2
Parang et al. [31], (Iran)	Nurses with MS (n = 30)	RCT, 8 weeks	Pilates vs. Control	Professional self-concept	2
Jiang et al. [18] (China)	Office workers (n = 64)	RCT, 6 weeks	Pilates vs. Neck stabilization	Pain, ROM, NDI, Posture (FHA/FSA)	1, 3
Stieglitz et al. [15] (Germany)	Hospital staff (n = 30)	OBS, 8 weeks	Pilates vs. Baseline	Pain (VAS), Disability (ODI)	1
Pérez Cunalata et al. [6] (Ecuador)	poultry workers (n = 130)	OBS, 6 months	Pilates vs. Baseline	Pain intensity, Flexibility	1
Barbosa & Oliveira [24,32] (Brazil)	Healthcare workers (n = 42)	RCT, 8 weeks	Pilates vs. Control	Depression, Anxiety, Occ health	2, 3
Tsai & Wang [14] (Taiwan)	Electronics workers (n = 34)	OBS, 8 weeks	Mat Pilates vs. Baseline	Leg strength, Abdominal endurance	1, 3
Alves et al. [33] (Brazil)	Workers with LBP (n = 32)	RCT, 8 weeks	Pilates vs. Traditional exercises	Pain, Disability, Kinesiophobia	1, 2
Dale et al. [34] (USA)	Office workers (n = 28)	RCT, 8 weeks	Pilates-based vs. Control	Pain, Grip strength, Function	1
Boix-Vilella et al. [22] (Spain)	Admin staff (n = 56)	OBS, 8 weeks	Pilates vs. Baseline	Emotional stability, Optimism	2
Kim et al. [7] (Korea)	Fruit farmers (n = 131)	OBS, 12 weeks	Prop Pilates vs. Baseline	Body stability, Pain	1, 3
Bulguroglu et al. [35] (Turkey)	Healthy adults (n = 58)	RCT, 8 weeks	Online vs. F2F Pilates vs. Control	Core endurance, Depression, QoL	1, 2, 3
Baniasadi et al. [26], (Iran)	COVID-19 Nurses (n = 30)	Q-EXP, 8 weeks	Pilates vs. Control	Anxiety, Job Stress	2
Tastan et al. [13] (Turkey)	Office workers (n = 22)	Q-EXP, 8 weeks	Reformer Pilates vs. Baseline	Posture, Body appreciation, Anxiety	1, 2
Bishe et al. [36] (Iran)	Female employees (n = 40)	Q-EXP, 8 weeks	On-the-job Pilates vs. Control	Job satisfaction	2, 3
Krawczky et al. [37] (Brazil)	Healthy adults (n = 37)	Q-EXP, 1 session	Pilates vs. Baseline	Postural alignment, Pain	1

Note: See Supplementary Table S1 for detailed study characteristics and key findings. OBS: Observational Study; RCT: Randomized Controlled Trial; Q-EXP: Quasi-experimental Study; wks: weeks; mths: months; CLBP: Chronic Low Back Pain; LBP: Low Back Pain; UCS: Upper Crossed Syndrome; MSD: Musculoskeletal Disorders; MS: Multiple Sclerosis; PT: Physical Therapy; MFR: Myofascial Release; PE: Postural Education; PNE: Pain Neuroscience Educa-tion; ROM: Range of Motion; NDI: Neck Disability Index; CVA: Craniovertebral Angle; FHA: Forward Head Angle; FSA: Forward Shoulder Angle; SAA: Social Appearance Anxiety; QoL: Quality of Life; QALY: Quality-Adjusted Life Year.

**Table 2:** Summary of Evidence by Pillar.

Study (Author, Year)	Pillar 1: Physical & Musculoskeletal Outcomes	Pillar 2: Mental & Psychological Outcomes	Pillar 3: Ergonomic & Occupational Outcomes
Guidotti et al. [20]	*Not evaluated*	Decrease: Anxiety, Depression, Somatization. Improvement: Stress management.	*Not evaluated*
Sabir et al. [28]	Decrease: Pain (NPRS), Disability (QBPDS).	*Not evaluated*	Improvement: Pelvic tilt angle.
Carregaro et al. [27]	*Not evaluated*	*Not evaluated*	Increase: QALYs. Decrease: Healthcare and societal costs (Dominant cost-effectiveness).
Hrusova & Komestik [29]	Improvement: Spine flexibility (Thomayer test only). Non-significant difference: Overall flexibility.	*Not evaluated*	*Not evaluated*
Karkousha et al. [19]	Decrease: Pain, Neck Disability (NDI).	*Not evaluated*	Improvement: Balance, Spinal curvature (CVA).
Kolomiitseva et al. [16]	Improvement: Respiratory function, Joint mobility, Muscle strength.	*Not evaluated*	*Not evaluated*
Maan et al. [25]	Decrease: Pain, Neck disability.	Decrease: Sleep disturbance (ISI).	Increase: Neck endurance.
Azam et al. [30]	Decrease: Pain.	Improvement: Quality of life (SF-12). Decrease: Kinesiophobia.	*Not evaluated*
Parang et al. [31]	*Not evaluated*	Improvement: Professional Self-Concept.	*Not evaluated*
Jiang et al. [18]	Decrease: Pain, Neck Disability (NDI). Improvement: Cervical ROM.	*Not evaluated*	Improvement: Postural alignment (FHA, FSA angles).
Stieglitz et al. [15]	Decrease: Pain (VAS), Disability (ODI).	*Not evaluated*	*Not evaluated*
Pérez Cunalata et al. [6]	Decrease: Pain intensity. Improvement: Muscle flexibility.	*Not evaluated*	*Not evaluated*
Barbosa et al. & Oliveira et al. [24,32]	*Not evaluated*	Decrease: Depression, Anxiety, Fatigue.	Improvement: Occupational health.
Tsai & Wang [14]	Increase: Lower limb strength.	*Not evaluated*	Increase: Abdominal endurance in the workplace.
Alves et al. [33]	Decrease: Pain, Disability.	Decrease: Catastrophizing, Fear-avoidance behavior.	*Not evaluated*
Dale et al. [34]	Decrease: Pain, Disability (PRTEE). Improvement: Grip strength.	*Not evaluated*	*Not evaluated*
Boix-Vilella et al. [22]	*Not evaluated*	Improvement: Emotional stability, Optimism.	*Not evaluated*
Kim et al. [7]	Decrease: Pain.	*Not evaluated*	Improvement: Body stability.
Bulguroglu et al. [35]	Increase: Core endurance.	Decrease: Depression. Improvement: Quality of life.	*Not evaluated*
Baniasadi et al. [26]	*Not evaluated*	Non-significant difference: Anxiety, Job stress.	*Not evaluated*
Taştan et al. [13]	*Not evaluated*	Improvement: Body appreciation. Decrease: Social appearance anxiety (SAA).	Improvement: Postural alignment.
Bishe et al. [36]	*Not evaluated*	*Not evaluated*	Increase: Job satisfaction.
Krawczky et al. [37]	Decrease: Pain.	*Not evaluated*	Improvement: Postural alignment.

Note: Two publications [24,32] were identified as companion reports deriving from the same randomized controlled trial. They were treated as a single study for the purpose of data extraction and synthesis to avoid double-counting the sample size. CVA, Craniovertebral Angle; FHA, Forward Head Angle; FSA, Forward Shoulder Angle; ISI, Insomnia Severity Index; NDI, Neck Disability Index; NPRS, Numeric Pain Rating Scale; ODI, Oswestry Disability Index; PRTEE, Patient-Rated Tennis Elbow Evaluation; QALYs, Quality-Adjusted Life Years; QBPDS, Quebec Back Pain Disability Scale; ROM, Range of Motion; SAA, Social Appearance Anxiety; SF-12, 12-Item Short Form Health Survey; VAS, Visual Analogue Scale.

**Table 3:** Intervention Characteristics of Included Pilates Studies.

Study (Author, Year)	Pilates Method & Equipment	Frequency & Session Length	Setting & Supervision
Guidotti et al. [20]	Matwork Pilates	Frequency not specified	Studio/Supervised
Sabir et al. [28]	Pilates Pelvic Tilt Exercises	3 sessions per week/Duration N/A	Clinical/Supervised
Carregaro et al. [27]	Mat Pilates with Accessories (Magic circle, ball, etc.)	2 sessions per week/50–60 min	Supervised Group Sessions
Hrusova & Komestik [29]	Modified Pilates Program	1 session per week + Home exercises	Mixed (Supervised & Unsupervised)
Karkousha et al. [19]	Mat Pilates Exercises	2 sessions per week/60 min	Physical Therapy Dept./Supervised
Kolomiitseva et al. [16]	Matwork Pilates	2 sessions per week/60 min	Supervised Training
Maan et al. [25]	Pilates with Breathing Re-education	3 sessions per week/60 min	Clinical Setting/Supervised
Azam et al. [30]	Mat Pilates Exercises	3 sessions per week/45–60 min	Clinical Setting/Supervised
Parang et al. [31]	Home-based Pilates	3 sessions per week/30 min	Home-based/Unsupervised
Jiang et al. [18]	Pilates Training + Fascial Massage	2 sessions per week/60 min	Supervised Studio
Stieglitz et al. [15]	Equipment-based (Reformer, Cadillac, Chair)	2 sessions per week/60 min	Occupational Med./Supervised
Pérez Cunalata et al. [6]	Pilates Exercise Program	3 sessions per week/Duration N/A	Workplace/Supervised
Barbosa et al. & Oliveira et al. [24,32]	Mat Pilates with Props (Ball, Flex band, Circle)	3 sessions per week/60 min	Workplace/Supervised
Tsai & Wang [14]	Polestar Pilates Method	2 sessions per week/60 min	Workplace/Supervised
Alves et al. [33]	Pilates + Postural Education	2 sessions per week/60 min	Supervised
Dale et al. [34]	Pilates-based Intervention	2 phases (Supervised + Home)	Mixed Supervision
Boix-Vilella et al. [22]	Matwork Pilates	Mean frequency: 2.82 days per week	Studio/Supervised
Kim et al. [7]	Prop Pilates Program (PPEP)	3 sessions per week/60 min	Community/Supervised
Bulguroglu et al. [35]	Online vs. Face-to-Face Mat Pilates	3 sessions per week/60 min	Supervised (Digital or F2F)
Baniasadi et al. [26]	Pilates Exercises	3 sessions per week/45–60 min	Workplace/Supervised
Taştan et al. [13]	Reformer Pilates	2 sessions per week/60 min	Supervised Studio
Bishe et al. [36]	On-the-job Pilates	3 sessions per week/30 min	Workplace/Supervised
Krawczky et al. [37]	Mat Pilates Exercises	Single session/30–40 min	Supervised

Note Two publications [24,32] are linked reports from the same clinical trial and are combined to ensure data consistency and avoid sample size inflation. F2F: Face-to-Face; N/A: Not Available; PPEP: Prop Pilates Exercise Program.

**Table 4:** Outcome Mapping: Measurement Tools Used Across Included Studies.

Study (Author, Year)	Pain Assessment	Functional Disability	Physical/Strength/Flexibility	Mental Health/Psychosocial	Occupational/Posture/Economics
Guidotti et al. [20]	-	-	-	SQ, PSQ	-
Sabir et al. [28]	NPRS	QBPDS	-	-	Pelvic Tilt Angle
Carregaro et al. [27]	-	-	-	-	EQ-5D-3L, Cost analysis
Hrusova & Komestik [29]	-	-	Thomayer, Schober, Stibor	-	-
Karkousha et al. [19]	VAS	NDI	Balance tests	-	CVA (Posture)
Kolomiitseva et al. [16]	-	-	Spirometry, Goniometry	-	-
Maan et al. [25]	VAS	NDI	Neck Endurance	ISI (Sleep)	-
Azam et al. [30]	VAS	-	-	SF-12, TSK	-
Parang et al. [31]	-	-	-	PSC Questionnaire	-
Jiang et al. [18]	VAS	NDI	Cervical ROM	-	FHA, FSA (Posture)
Stieglitz et al. [15]	VAS	ODI	-	-	-
Pérez Cunalata et al. [6]	Pain Intensity	-	Sit-and-Reach	-	-
Barbosa et al., & Oliveira et al., [24,32]	-	-	-	BDI, STAI, FAS	Occ Health Scale
Tsai & Wang [14]	-	-	Leg strength tests	-	Abdominal Endurance
Alves et al. [33]	NRS	ODI	Back extension test	PCS, FABQ	-
Dale et al. [34]	PRTEE (Pain)	PRTEE (Func)	Grip Strength	-	-
Boix-Vilella et al. [22]	-	-	-	NEO-FFI, LOT-R	-
Kim et al. [7]	Pain Scale	-	Body Stability	-	-
Bulguroglu et al. [35]	-	-	Core Endurance	BDI, SF-36	-
Baniasadi et al. [26]	-	-	-	Anxiety & Job Stress Scales	-
Taştan et al. [13]	-	-	NY Posture Scale	BAS, SAAS	Posture assessment
Bishe et al. [36]	-	-	-	-	Job Satisfaction Scale
Krawczky et al. [37]	Pain Scale	-	SAPO (Posture angles)	-	Postural alignment

Note: BAS: Body Appreciation Scale; BDI: Beck Depression Inventory; CVA: Craniovertebral Angle; FABQ: Fear-Avoidance Beliefs Questionnaire; FAS: Fatigue Assessment Scale; HADS: Hospital Anxiety and Depression Scale; NDI: Neck Disability Index; NPRS: Numerical Pain Rating Scale; ODI: Oswestry Disability Index; PCS: Pain Catastrophizing Scale; PSQ: Psychosomatic Stress Questionnaire; QBPDS: Quebec Back Pain Disability Scale; SAAS: Social Appearance Anxiety Scale; SF-12/36: Short Form Health Surveys; SQ: Symptom Questionnaire; STAI: State–Trait Anxiety Inventory; TSK: Tampa Scale for Kinesiophobia; VAS: Visual Analogue Scale.

**Table 5:** Risk of Bias Assessment. (**a**). Randomized Controlled Trials (RoB 2 Tool); (**b**). Non-Randomized Studies (ROBINS-I Tool).

(**a**)								
**Study (Author, Year)**	**D1**	**D2**		**D3**	**D4**		**D5**	**Overall Risk of Bias**
Sabir et al. [28]	!	!		+	!		+	Some Concerns
Carregaro et al. [27]	+	+		+	+		+	Low Risk
Karkousha et al. [19]	+	+		+	+		+	Low Risk
Kolomiitseva et al. [16]	!	!		+	!		!	Some Concerns
Maan et al. [25]	+	!		+	+		+	Low Risk
Azam et al. [30]	!	!		+	!		!	Some Concerns
Parang et al. [31]	!	!		+	!		!	Some Concerns
Jiang et al. [18]	!	!		+	!		+	Some Concerns
Barbosa et al. [24]	!	!		-	!		!	High Risk
Alves et al. [33]	!	!		+	!		+	Some Concerns
Dale et al. [34]	!	!		+	!		!	Some Concerns
Bulguroglu et al. [35]	+	!		+	+		+	Low Risk/Some Concerns
(**b**)								
**Study (Author, Year)**	**D1**	**D2**	**D3**	**D4**	**D5**	**D6**	**D7**	**Overall Risk of Bias**
Guidotti et al. [20]	-	!	+	!	+	!	+	Serious Risk
Hrusova & Komestik [29]	x	!	+	+	+	!	+	Critical Risk
Stieglitz et al. [15]	x	!	+	!	+	!	!	Critical Risk
Pérez Cunalata et al. [6]	x	!	+	!	+	!	!	Critical Risk
Tsai & Wang [14]	!	!	+	!	+	!	+	Moderate Risk
Boix-Vilella et al. [22]	-	-	+	!	+	-	!	Serious Risk
Kim et al. [7]	x	!	+	!	+	!	+	Critical Risk
Baniasadi et al. [26]	-	!	+	!	+	!	+	Serious Risk
Taştan et al. [13]	x	!	+	!	+	!	+	Critical Risk
Bishe et al. [36]	-	!	+	!	+	!	+	Serious Risk
Krawczky et al. [37]	x	!	+	!	+	!	+	Critical Risk

Note for RoB 2 (RCTs): D1: Bias arising from the randomization process; D2: Bias due to deviations from intended interventions; D3: Bias due to missing outcome data; D4: Bias in measurement of the outcome; D5: Bias in selection of the reported result. (+): Low Risk; (!): Some Concerns; (-): High Risk. Note for ROBINS-I (Non-RCTs): D1: Bias due to confounding; D2: Bias in selection of participants into the study; D3: Bias in classification of interventions; D4: Bias due to deviations from intended interventions; D5: Bias due to missing data; D6: Bias in measurement of outcomes; D7: Bias in selection of the reported result. (+): Low Risk; (!): Moderate Risk; (-): Serious Risk; (x): Critical Risk.

**Table 6:** GRADE Summary of Findings (Pilates vs. Control in Workers—Only RCTs).

Outcome	Population (RCTs)	Effect (Pilates vs. Control)	Certainty (GRADE)	Comments
Pain	Workers with chronic low back or neck pain (8 RCTs)	Pilates probably reduces pain in the short term	Moderate	Consistent direction of benefit; downgraded for risk of bias and imprecision.
Functional Disability	Workers with CLBP or neck pain (6 RCTs)	Pilates probably improves disability	Moderate	Improvements across studies; downgraded for risk of bias and imprecision.
Psychological Outcomes (anxiety, depression, stress, kinesiophobia, catastrophizing, sleep)	Workers with musculoskeletal pain or healthcare workers (2 RCTs)	Pilates may reduce psychological distress	Low	Few RCTs; heterogeneous measures; one high-risk study; downgraded for RoB and imprecision.
QALYs/Cost-Effectiveness	Workers with chronic low back pain (1 RCT)	Pilates probably improves QALYs and is cost-effective	Moderate	Single high-quality RCT; downgraded for imprecision.

## Data Availability

No new data were created or analyzed in this study.

## Supplementary Materials

The following supporting information can be downloaded at https://www.mdpi.com/article/10.3390/healthcare14131852/s1, Table S1: Detailed Characteristics of Included Studies; Table S2: Risk of Bias Summary for Randomized Controlled Trials (RoB 2 Tool); Table S3: Expanded GRADE Evidence Profile for Pilates Interventions in Workers.

## Abbreviations

The following abbreviations are used in this manuscript:

PICOS	Population, Intervention, Comparator, Outcome, Study design
WMSDs/WRMSDs	Work-Related Musculoskeletal Disorders
CLBP	Chronic Low Back Pain
UCS	Upper Cross Syndrome
RCTs	Randomized Controlled Trials
ROBINS-I	Risk Of Bias In Non-randomized Studies—of Interventions
RoB 2	Risk of Bias 2
QALYs	Quality-Adjusted Life Years
VAS	Visual Analogue Scale
NDI	Neck Disability Index
ODI	Oswestry Disability Index
ROM	Range of Motion
FHA/FSA	Forward Head Angle/Forward Shoulder Angle
QoL	Quality of Life
F2F	Face-to-Face
Int$	International Dollars

## Appendix A

### Search Strategies and Syntax per Platform

PubMed (Search conducted on 30 April 2026).

Query String: ((“Pilates”[Mesh] OR “Pilates”[Title/Abstract] OR “Pilates method”[Title/Abstract] OR “Reformer Pilates”[Title/Abstract] OR “Mat Pilates”[Title/Abstract])) AND (“workplace”[Title/Abstract] OR “occupational health”[Title/Abstract] OR “corporate wellness”[Title/Abstract] OR “office workers”[Title/Abstract] OR “healthcare staff”[Title/Abstract] OR “remote workers”[Title/Abstract]) AND (“musculoskeletal”[Title/Abstract] OR “burnout”[Title/Abstract] OR “ergonomics”[Title/Abstract] OR “productivity”[Title/Abstract]).

Filters applied: None (No language or publication date restrictions were selected in the database interface to maximize sensitivity).

Yield: n = 144 records.

Scopus (Search conducted on 30 April 2026).

Query String: (TITLE-ABS-KEY(“Pilates”) OR TITLE-ABS-KEY(“Pilates method”) OR TITLE-ABS-KEY(“Reformer Pilates”) OR TITLE-ABS-KEY(“Mat Pilates”)) AND (TITLE-ABS-KEY(“workplace”) OR TITLE-ABS-KEY(“occupational health”) OR TITLE-ABS-KEY(“corporate wellness”) OR TITLE-ABS-KEY(“office workers”) OR TITLE-ABS-KEY(“healthcare staff”) OR TITLE-ABS-KEY(“remote workers”)) AND (TITLE-ABS-KEY(“musculoskeletal”) OR TITLE-ABS-KEY(“burnout”) OR TITLE-ABS-KEY(“ergonomics”) OR TITLE-ABS-KEY(“productivity”)).

Filters applied: None.

Yield: n = 211 records.

Web of Science (Meta-search Engine—Search conducted on 30 April 2026).

Query String: TS = (“Pilates” OR “Pilates method” OR “Reformer Pilates” OR “Mat Pilates”) AND TS = (“workplace” OR “occupational health” OR “corporate wellness” OR “office workers” OR “healthcare staff” OR “remote workers”) AND TS = (“musculoskeletal” OR “burnout” OR “ergonomics” OR “productivity”).

Filters applied: None (All citation indexes selected; no language filters applied).

Yield: n = 123 records.

PEDro (Physiotherapy Evidence Database—Search conducted on 30 April 2026).

Note: Due to interface limitations regarding complex Boolean operators, combinations of terms were searched individually using the advanced search fields.

Query combination examples: * Abstract & Title: Pilates AND Subdiscipline: occupational health.

Abstract & Title: Pilates AND Therapy: fitness training.

Filters applied: None.

Yield: n = 57 records.

Google Scholar (Meta-search Engine/Grey Literature Source—Search conducted on 30 April 2026).

Query String: with all of the words: Pilates | with at least one of the words: workplace “occupational health” “corporate wellness” “office workers”.

Search constraints: Sorting by relevance was applied, and the first 300 records were screened sequentially until theoretical saturation was reached (no further unique relevant studies appeared).

Yield: n = 328 records.

Note on Language Filters (Addressing Reviewer 3):

No language restriction filters were activated in any database or search engine setup. The search terms were entered in English to capture international literature indexed with English metadata (titles, abstracts, keywords). Non-English language papers identified during the screening process were eligible for translation; however, all final meeting the inclusion criteria studies were published in English.
