# Supplementary material for: Pilates Method as a Biopsychosocial Intervention in the Modern Workplace: A Systematic Review of Physical, Mental, and Occupational Benefits
Source: Healthcare (Basel). 2026 Jun 25;14(13):1852. doi: 10.3390/healthcare14131852 (PMC13361879; doi:10.3390/healthcare14131852)
Supplement: Supplementary file 1 [file healthcare-14-01852-s001.zip › Supplementary Table S1. Detailed Characteristics of Included Studies.pdf]

Supplementary Table S1. Detailed Characteristics of Included Studies

Study (Year, Country)	Population / Occupation	Sample Size	Design	Intervention vs Comparator	Duration	Outcomes	Key Findings	Pillar
**Guidotti et al. (2025, Italy)**	Adults 50–64 years old	n=49 (25 Pilates, 24 Control)	Observational study	Pilates Matwork vs Non-active controls	12 weeks	Psychological symptoms (SQ), Stress risk behaviors (PSQ)	Significant reductions in anxiety, depression, somatization; improved stress management.	2
**Sabir et al. (2024, Pakistan)**	Power loom workers with chronic low back pain	n=46 (23 per group)	Randomized Controlled Trial (RCT)	Pilates Pelvic Tilt vs McKenzie extension	4 weeks	Pain (NPRS), Disability (QBPDS), Pelvic tilt angle	Both effective, but Pilates was superior in reducing pain, disability, and pelvic	1 & 3

Study (Year, Country)	Population / Occupation	Sample Size	Design	Intervention vs Comparator	Duration	Outcomes	Key Findings	Pillar
							tilt angle.	
**Carregaro et al. (2024, Brazil)**	Adults with chronic non-specific low back pain	n=145 (72 Pilates, 73 Home-based)	RCT with economic evaluation	Group-based Pilates vs Home-based exercises	6 weeks	QALYs, Healthcare and societal costs	Pilates was found to be more cost-effective and dominant from a societal perspective.	3
**Hrusova & Komestik (2014, Czech Republic)**	Sedentary female workers	n=21	Quasi-experimental (pre-post)	Modified Pilates programme vs Baseline	3 months	Spine flexion (Thomayer, Schober, Stibor)	Improvement only in Thomayer test; no overall significant difference in general spinal flexibilit	1

Study (Year, Country)	Population / Occupation	Sample Size	Design	Intervention vs Comparator	Duration	Outcomes	Key Findings	Pillar
							y.	
**Karkousha et al. (2024, Egypt)**	Females with Upper Crossed Syndrome	n=40 (20 per group)	Double-blind RCT	Pilates vs Traditional physical therapy	4 weeks	Spinal curvature, Craniovertebral angle (CVA), Balance, Pain, NDI	Pilates produced significantly greater improvements in posture, balance, CVA, and pain.	1 & 2
**Kolomiitseva et al. (2022, Ukraine)**	Middle-aged women with sedentary occupations	n=32 (18 Pilates, 14 Control)	Randomized controlled study	Pilates training vs No exercise	12 weeks	Respiratory function, joint mobility, muscle strength	Significant improvements in respiratory function, joint mobility, and abdominal/shoulder strength.	1

Study (Year, Country)	Population / Occupation	Sample Size	Design	Intervention vs Comparator	Duration	Outcomes	Key Findings	Pillar
**Maan et al. (2026, Pakistan)**	Working women with chronic non-specific neck pain	n=52	Parallel-group RCT	Pilates + Breathing re-education vs Pilates alone	6 weeks	Pain (NPRS), Neck endurance, Sleep disturbance (ISI), Disability	Combined program superior to Pilates alone across all tested variables.	1 & 2
**Azam et al. (2022, Pakistan)**	Female office workers with chronic non-specific neck pain	n=70	RCT	Pilates vs Neck stabilization exercises	6 weeks	Pain (NPRS), Quality of life (SF-12), Kinesiophobia	Both groups improved significantly; no significant difference between the two interventions.	1 & 2
**Parang et al. (2020,**	Nurses in ICUs &	n=110	Randomized controlle	Home-based Pilates	8 weeks	Professional Self-	Significant improve	2

Study (Year, Country)	Population / Occupation	Sample Size	Design	Intervention vs Comparator	Duration	Outcomes	Key Findings	Pillar
**Iran)**	Emergency Departments		d field trial	vs Control		Concept (PSC)	ment in total PSC and several subdomains in the Pilates group.	
**Jiang et al. (2025, Thailand)**	Office workers with Upper Crossed Syndrome	n=34	RCT	Pilates + fascial massage vs Pilates alone	12 weeks	FHA, FSA, cervical ROM, sEMG, pain (VAS), NDI	Combined program produced greater improvements in posture, ROM, and muscle activation than Pilates alone.	1 & 2
**Stieglitz et al. (2016, USA)**	Workers with work-related chronic	n=12	Quasi-experimental pilot	Supervised equipment-based Pilates	6 weeks	Pain (VAS), Disability (ODI)	Significant reductions in pain and	1 & 3

Study (Year, Country)	Population / Occupation	Sample Size	Design	Intervention vs Comparator	Duration	Outcomes	Key Findings	Pillar
	low back pain			vs Baseline			disability with large effect sizes.	
**Cunalata et al. (2024, Ecuador)**	Poultry workers with MSDs	n=29	Quasi-experimental	Pilates-based exercise vs Baseline	12 weeks	Flexibility, Pain intensity	Significant improvements across the population in reducing pain and increasing flexibility.	1
**Fleming et al. (2021, Ireland)**	Persons with Multiple Sclerosis (home-based)	n=80	RCT	Home-based Pilates vs Control	8 weeks	Anxiety, Depression, Fatigue	Home-based Pilates significantly reduced symptoms of anxiety, depressi	2

Study (Year, Country)	Population / Occupation	Sample Size	Design	Intervention vs Comparator	Duration	Outcomes	Key Findings	Pillar
							on, and fatigue.	
**Barbosa et al. (2018) & Oliveira et al. (2018) [Same cohort, Brazil]**	Banking sector workers / Bank employees	n=50	Clinical, controlled, randomized, blind study	Pilates vs Quick massage vs Control	9 months	Depression, Anxiety, Fatigue, Occupational health	Both Pilates and massage reduced depression, anxiety, and fatigue and positively influenced occupational health.	2 & 3
**Tsai & Wang (2016, Taiwan)**	Workplace employees	n=88	Quasi-experimental	Workplace Pilates vs No exercise	12 weeks	Lower limb strength, Abdominal endurance	Significant enhancement in lower limb strength and abdomin	1 & 3

Study (Year, Country)	Population / Occupation	Sample Size	Design	Intervention vs Comparator	Duration	Outcomes	Key Findings	Pillar
							al endurance in the workplace.	
**Alves et al. (2024, Portugal)**	Paper industry workers with LBP	n=37	Pilot RCT	Pilates + Postural education vs PNE + Graded exposure	8 weeks	Pain, disability, catastrophizing, fear-avoidance	Pilates reduced pain similarly to PNE, but PNE was superior for psychosocial factors.	1 & 3
**Dale et al. (2016, USA)**	Workers with lateral epicondylosis (Tennis Elbow)	n=17	Pilot RCT	Pilates + Standard therapy vs Standard therapy alone	6-8 weeks	Pain, disability (PRTEE), grip strength	Pilates group showed greater improvements, though not statistically significantly	1

Study (Year, Country)	Population / Occupation	Sample Size	Design	Intervention vs Comparator	Duration	Outcomes	Key Findings	Pillar
							different from control.	
**Boix-Vilella et al. (2017, Spain)**	Service sector workers	n=63	Descriptive-comparative study	Pilates vs Sedentary (no exercise)	Cross-sectional	Psychosocial health (emotional stability, optimism)	The Pilates group recorded higher levels of emotional stability and optimism.	2
**Kim et al. (2014, Korea)**	Fruit farmers with MSDs	n=131	Quasi-experimental	Prop Pilates vs Baseline	12 weeks	Body stability, Pain	Significant improvements in body stability in both male and female farmers.	1 & 3
**Bulguro**	Healthy	n=58	Random	Online	8 weeks	Core	Both	1, 2 & 3

Study (Year, Country)	Population / Occupation	Sample Size	Design	Intervention vs Comparator	Duration	Outcomes	Key Findings	Pillar
**glu et al. (2023, Turkey)**	individuals (COVID-19 pandemic)		ized controlled study	Pilates vs Face-to-Face Pilates vs Control		endurance, Depression, QoL	online and face-to-face Pilates significantly improved core endurance, depression, and QoL.	
**Baniasadi et al. (2022, Iran)**	COVID-19 Nurses	n=?	Quasi-experimental	Pilates vs Control	8 weeks	Anxiety, Job Stress	Pilates alone did not significantly affect anxiety and job stress without other interventions.	2
**Taştan et al.**	Desk-based	n=22	Quasi-experim	Reformer Pilates	8 weeks	Postural alignme	Significant	1 & 2

Study (Year, Country)	Population / Occupation	Sample Size	Design	Intervention vs Comparator	Duration	Outcomes	Key Findings	Pillar
**(2025, Turkey)**	office workers		ental	vs Baseline		nt, body appreciation, social appearance anxiety	improvements in posture, body appreciation, and reductions in social anxiety.	
**Bishe et al. (2019, Iran)**	Female employees	n=?	Quasi-experimental	On-the-job Pilates vs Control	8 weeks	Job satisfaction	On-the-job Pilates significantly improved job satisfaction among female employees.	2 & 3
**Krawczky et al. (2016, Brazil)**	Healthy adults	n=?	Quasi-experimental	Pilates vs Baseline	Single session	Postural alignment, Pain	Improved postural alignment in the sagittal	1

Study (Year, Country)	Population / Occupation	Sample Size	Design	Intervention vs Comparator	Duration	Outcomes	Key Findings	Pillar
							plane following a single session.	
