# Supplementary material for: Pilates Method as a Biopsychosocial Intervention in the Modern Workplace: A Systematic Review of Physical, Mental, and Occupational Benefits
Source: Healthcare (Basel). 2026 Jun 25;14(13):1852. doi: 10.3390/healthcare14131852 (PMC13361879; doi:10.3390/healthcare14131852)
Supplement: Supplementary file 1 [file healthcare-14-01852-s001.zip › Supplementary Table S2. Risk of Bias Summary for Randomized Controlled Trials (RoB 2 Tool).pdf]

Supplementary Table S2. Risk of Bias Summary for Randomized Controlled Trials (RoB 2 Tool)

Study (Author, Year)	D1: Randomization Process	D2: Deviations from Intended Interventions	D3: Missing Outcome Data	D4: Measurement of the Outcome	D5: Selection of the Reported Result	Overall Risk of Bias
**Sabir et al. (2024)**	Some concerns	Some concerns	Low risk	Some concerns	Low risk	**Some concerns**
**Carregaro et al. (2024)**	Low risk	Low risk	Low risk	Low risk	Low risk	**Low risk**
**Karkousha et al. (2024)**	Low risk	Low risk	Low risk	Low risk	Low risk	**Low risk**
**Kolomiitseva et al. (2022)**	Some concerns	Some concerns	Low risk	Some concerns	Some concerns	**Some concerns**
**Maan et al. (2026)**	Low risk	Some concerns	Low risk	Low risk	Low risk	**Low risk**
**Azam et al. (2022)**	Some concerns	Some concerns	Low risk	Some concerns	Some concerns	**Some concerns**
**Parang et al. (2020)**	Some concerns	Some concerns	Low risk	Some concerns	Some concerns	**Some concerns**
**Jiang et al. (2025)**	Some concerns	Some concerns	Low risk	Some concerns	Low risk	**Some concerns**
**Barbosa et al. (2018)**	Some concerns	Some concerns	High risk	Some concerns	Some concerns	**High risk**
**Alves et al. (2024)**	Some concerns	Some concerns	Low risk	Some concerns	Low risk	**Some concerns**
**Dale et al. (2016)**	Some concerns	Some concerns	Low risk	Some concerns	Some concerns	**Some concerns**
**Bulguroglu et al. (2023)**	Low risk	Some concerns	Low risk	Low risk	Low risk	**Low risk / Some concerns**
