# Supplementary material for: Pilates Method as a Biopsychosocial Intervention in the Modern Workplace: A Systematic Review of Physical, Mental, and Occupational Benefits
Source: Healthcare (Basel). 2026 Jun 25;14(13):1852. doi: 10.3390/healthcare14131852 (PMC13361879; doi:10.3390/healthcare14131852)
Supplement: Supplementary file 1 [file healthcare-14-01852-s001.zip › Supplementary Table S3. Expanded GRADE Evidence Profile for Pilates Interventions in Workers..pdf]

Supplementary Table S3. Expanded GRADE Evidence Profile

Outcome	Study Design	Risk of Bias	Inconsistency	Indirectness	Imprecision	Publication Bias	Overall Certainty	Explanation
Pain	8 RCTs	Serious	Not serious	Not serious	Serious	Undetected	Moderate	Several RCTs had “some concerns” in randomization and outcome measurement; small sample sizes contributed to imprecision. Effects were consistently in favor of Pilates.
Functional Disability	6 RCTs	Serious	Not serious	Not serious	Serious	Undetected	Moderate	Improvements were consistent across studies. Downgrading was applied for risk of bias and small sample sizes.
Psychological Outcomes	2 RCTs	Serious	Serious	Not serious	Serious	Undetected	Low	Only two RCTs contributed data; one had high risk of bias.

Outcome	Study Design	Risk of Bias	Inconsistency	Indirectness	Imprecision	Publication Bias	Overall Certainty	Explanation
								Heterogeneous psychological measures and small samples increased inconsistency and imprecision.
QALYs / Cost-Effectiveness	1 RCT	Not serious	Not applicable	Not serious	Serious	Undetected	Moderate	Evidence derived from a single high-quality RCT. Downgraded for imprecision due to single-study evidence.
